# Traditional Korean Medicine Services and Its Association with Knee Surgery and Opioid Use in Patients with Knee Osteoarthritis: A Nationwide Retrospective Study in Korea

**DOI:** 10.3390/jcm14207152

**Published:** 2025-10-10

**Authors:** Hwang Woo Seok, Ho-Yeon Go, Won-Hyung Ryu, Yoon Jae Lee, In-Hyuk Ha, Doori Kim

**Affiliations:** 1 Jaseng Hospital of Korean Medicine, Seoul 06110, Republic of Korea; hpsuk2824@naver.com; 2 Department of Korean Internal Medicine, Semyung University, Jecheon-si 27136, Republic of Korea; kohoyeon@gmail.com; 3 Mokdong Jaseng Hospital of Korean Medicine, Seoul 08023, Republic of Korea; rwh0225@naver.com; 4 Jaseng Spine and Joint Research Institute, Jaseng Medical Foundation, Seoul 06110, Republic of Korea; goodsmile8119@gmail.com (Y.J.L.); hanihata@gmail.com (I.-H.H.)

**Keywords:** Korean medicine, traditional Korean medicine services, knee osteoarthritis, opioid use, knee surgery

## Abstract

**Objectives**: This study aimed to assess the impact of traditional Korean medicine services (TKMS) on subsequent knee surgery and opioid use in patients diagnosed with knee osteoarthritis (KOA). **Methods**: This retrospective cohort study used National Health Insurance Review and Assessment Service claims data from 2015 to 2017 to identify patients treated for KOA (M17) in 2016. Patients with at least two Korean medicine (KM) clinic visits within 6 weeks of the initial diagnosis formed the TKMS group, while those without visits to KM clinics formed the n group. Propensity score matching (PSM) (1:1) was applied and the incidence of knee surgery and opioid use was followed up for one year. Kaplan–Meier survival curves and Cox proportional hazards models estimated time-to-event outcomes and hazard ratios (HRs). Sensitivity analyses were performed to verify the results across varied treatment windows of 4, 8, and 10 weeks. **Results**: After PSM, 247,168 patients were included in the analysis for each group. The TKMS group exhibited significantly lower HRs for knee surgery (HR = 0.69, 95% CI: 0.66–0.72), opioid use (HR = 0.66, 95% CI: 0.65–0.66), and their compound events (HR = 0.66, 95% CI: 0.65–0.67) compared with the Non-TKMS group. The results remained consistent across sensitivity analyses. **Conclusions**: Among patients with KOA, the utilization of TKMS may significantly reduce the incidence of knee surgery and opioid use. Thus, the utilization of TKMS may be associated with a reduced need for unnecessary surgical interventions and with lower reliance on high-risk medications.

## 1. Introduction

Knee osteoarthritis (KOA) is a common condition that affects approximately 30% of individuals aged ≥45, characterized by cartilage degradation, bone remodeling, osteophyte formation, joint inflammation, and impaired joint function due to abnormal joint metabolism [1]. KOA represents approximately 80% of global osteoarthritis cases, with its incidence rising alongside obesity and age [2]. In South Korea, KOA is the leading disease among adults ≥65 years, and associated surgeries rank first in terms of costs [3,4], highlighting its significant socioeconomic impact.

The primary goal of treating osteoarthritis is to alleviate symptoms and slow disease progression. Current recommendations for KOA include physical therapy, orthopedic aids, and pharmacotherapy. Surgery is considered when all conservative measures fail [4]. Among nonsurgical treatments, nonsteroidal anti-inflammatory drugs (NSAIDs), opioids, and glucocorticoids are most commonly used. From 2000 to 2015 in the UK, 84% of patients with KOA were prescribed opioids [5]. However, the 2021 American Academy of Orthopedic Surgeons (AAOS) Clinical Practice Guidelines on the Management of KOA notes that oral opioids offer limited pain relief or function improvement and carry significant risk of side effects [6]. Typical adverse effects related to opioid therapy comprise sedation, dizziness, nausea, vomiting, constipation, physical dependence, tolerance, and respiratory depression [7]. Therefore, cautious use of opioids is warranted.

Knee arthroplasty is considered the standard surgical intervention for KOA. Although it remains an effective option, it is associated with high costs [8], an increased risk of postoperative infection, and a likelihood that younger patients may require subsequent revision surgeries [9]. Additionally, certain patients may have contraindications due to individual characteristics, warranting careful consideration when designing treatment regimens [10].

Recent analyses of global research trends in nonsurgical treatments for KOA indicate a declining proportion of studies focusing on pharmacotherapy, alongside growing interest in physical therapy and complementary or alternative therapies, such as acupuncture [11]. South Korea operates a dual healthcare system consisting of Western medicine service and traditional Korean medicine service (TKMS), where patients may choose between the two according to their symptoms and preferences [12]. In this context, “TKMS” refers to services provided by licensed Korean medicine (KM) physicians in KM clinics or hospitals, which typically include modalities such as acupuncture, electroacupuncture, pharmacopuncture, Chuna manual therapy, moxibustion, and cupping [12].

Particularly, KOA is one of the conditions for which patients frequently seek TKMS. In 2023, approximately 480,000 patients with KOA utilized TKMS in South Korea [3]. In the global context, other complementary and integrative medicine (CIM) approaches are also used for KOA management, such as Ayurvedic therapies [13] in South Asia, and osteopathic [14] or chiropractic manual therapies [15] in western countries. Within this landscape, KM represents a nationally institutionalized CIM system, integrated into the dual healthcare structure of South Korea and characterized by the delivery of multimodal interventions by licensed physicians.

Evidence supporting the efficacy of TKMS for KOA has been steadily accumulating. A randomized controlled trial (RCT) involving patients with chronic KOA of Kellgren–Lawrence grade 2 or lower demonstrated that acupuncture significantly improved the Western Ontario and McMaster Universities Osteoarthritis Index (WOMAC) scores compared with a waiting-list control [16]. In addition, moxibustion was reported to provide greater improvements in pain and function than usual treatments [17], and electroacupuncture showed superior benefits compared with pharmacotherapy [18]. An observational study of 180 inpatients who received TKMS—including acupuncture, herbal medicine, and pharmacopuncture—reported significant improvements in pain, functional indices, and quality of life, with 96% of patients indicating clinical improvement and no adverse events observed [19]. Collectively, these findings suggest that TKMS may serve as an effective and safe treatment option for patients with KOA.

However, while research on acupuncture has been relatively active, fewer studies have investigated other KM modalities such as herbal medicine, pharmacopuncture, or electroacupuncture. In clinical practice in South Korea, patients generally receive not a single modality but a combination of KM interventions; to our knowledge, only one small-scale observational study has reported the effects of such integrative KM interventions [19]. This discrepancy underscores the need for studies assessing the overall impact of TKMS as delivered in real-world multimodal practice. Furthermore, despite the well-known limitations of opioid and surgical treatments including tolerance, side effects, and high costs [7,10], no prior study has examined whether TKMS may mitigate these risks or explored the associations between TKMS with opioid use or surgical interventions. Considering the safety and cost concerns of opioids and surgery, clarifying whether TKMS can reduce reliance on these treatments is of particular clinical and policy relevance.

Therefore, in this study, we utilized customized data from the Health Insurance Review and Assessment Service (HIRA) to investigate the association between TKMS and the risks of knee surgery and opioid use in patients with KOA. We hypothesized that the utilization of TKMS would be associated with a reduced risk of undergoing surgery and using opioids compared with those who did not utilize TKMS.

## 2. Materials and Methods

### 2.1. Data Source

This study used a customized dataset from the Health Insurance Review and Assessment Service (HIRA) database (2015–2017). HIRA collects basic demographic details, medical service records, and expense information for each patient in South Korea, based on claims submitted by healthcare providers to the National Health Insurance Service. Researchers can request customized datasets tailored to their studies, which include data from all medical service bills for the entire Korean population. Data requests are handled via the HIRA Open Data Portal [20].

Diagnoses were coded using the Korean Standard Classification of Diseases, Version 7 (KCD-7), based on the International Classification of Diseases, 10th Revision Clinical Modification (ICD-10-CM) [19,21]. Researchers received data with encrypted personal identification numbers to protect privacy. Analyses can be performed remotely at a designated center, with only result tables exportable.

This study was approved by the Institutional Review Board (IRB) of the Jaseng Hospital of Korean Medicine (IRB Approval No.: JASENG 2024-11-011, Date of Approval: 29 November 2024). Patient consent was waived because the analysis was conducted using de-identified and encrypted data, making it impossible to obtain consent from individual patients, and the study posed no risk of harm to patients.

### 2.2. Study Population

**Figure 1:**
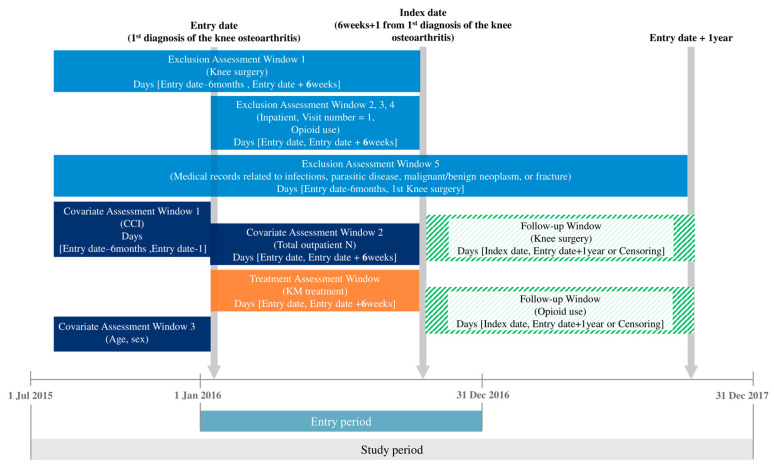
Study design and assessment windows.

The study population comprised patients who used medical services with KOA as the primary diagnosis in 2016. KOA was defined using the KCD-7 code M17. The entry date was set to be the date of the first visit to a medical institution with osteoarthritis of knee as the primary diagnosis in 2016. To ensure baseline homogeneity of the study population, a 6-month washout period was applied; patients who underwent knee surgery or were diagnosed with malignancies during this period were excluded. Additional exclusion criteria were as follows: patients who had knee surgery, hospital admission, or opioid prescriptions with an M17 diagnosis as the primary or secondary diagnosis within 6 weeks from the entry date; and patients who used medical services for parasitic or infectious diseases, neoplasms, fractures, or infectious arthropathies during the study period from the entry date (Figure 1).

### 2.3. Intervention

The study population was divided into TKMS and Non-TKMS groups. The TKMS group included patients who used TKM two or more times within 6 weeks of the entry date, with KOA as the primary or secondary diagnosis. TKMS was defined as having claims records of visits to KM clinics or hospitals, regardless of the specific modalities received. Detailed treatment information, such as acupuncture or cupping, was not considered in this classification. The Non-TKMS group consisted of patients who did not visit KM institutions within 6 weeks of the entry date but had the same diagnosis criteria.

### 2.4. Outcome Measures

Three outcomes were measured: knee surgery, opioid use, and either knee surgery or opioid use. Knee surgery included total knee replacement, partial knee replacement, revision knee replacement, knee osteotomy, knee arthroplasty, arthrodesis, meniscectomy, meniscal suture, meniscal transplantation, and cruciate ligament reconstruction and repair. Opioid use was defined as having at least one prescription for medications classified under anatomical therapeutic chemical (ATC) code N02A. The ATC system, overseen by the WHO, is an international standard that categorizes active substances according to the organ or body system on which they act and their therapeutic, pharmacological, and chemical properties [22]. In this system, cod N02A specifically refers to opioids [23]. The procedure codes for knee surgeries are summarized in Supplementary Table S1. The follow-up window covered knee surgery and opioid use.

The composite outcome of “either knee surgery or opioid use” was included to capture overall treatment escalation, as both events represent high-risk or invasive management strategies in KOA. This composite endpoint allowed for a more comprehensive assessment of the potential association between TKMS utilization and the reduced need for such treatments.

### 2.5. Statistical Analysis

Propensity score matching (1:1) was performed based on age, sex, Charlson comorbidity index (CCI), and the number of outpatient visits over a 6-week period. The CCI was calculated based on medical records from the 6 months preceding the entry date. Outpatient visits for relevant diagnoses (Korean and Western medicine) were summed and grouped into three categories: 2–5, 6–11, and ≥12 visits. Baseline characteristics after propensity score matching were compared using McNemar’s test and standardized mean differences (SMDs) [24].

Survival analysis was performed, with time to knee surgery, opioid use, and compound events of knee surgery or opioid use plotted using Kaplan–Meier curves [25]. The log-rank test was used to assess differences between groups. Cox proportional hazards regression was used to calculate hazard ratios (HRs) and 95% confidence intervals (CIs) for knee surgery and opioid use. When calculating HRs, sex, age, CCI, and number of outpatient visits served as covariates for adjustment in sensitivity analyses. Before performing Cox proportional, the proportional hazards (PHs) assumption was evaluated along log–log plots.

Sensitivity analyses classified groups based on whether patients accessed TKMS (visiting KM institutions) within 4-, 8-, and 10-week TKMS treatment windows, instead of the standard 6 weeks. These time frames were determined through internal discussion among the research team. For each window, event incidence (knee surgery, opioid use, or knee surgery or opioid use) and HRs were calculated. All statistical analyses were performed using the SAS statistical software suite (SAS 9.4; SAS Institute, Inc., Cary, NC, USA).

## 3. Results

### 3.1. Baseline Statistics

**Figure 2:**
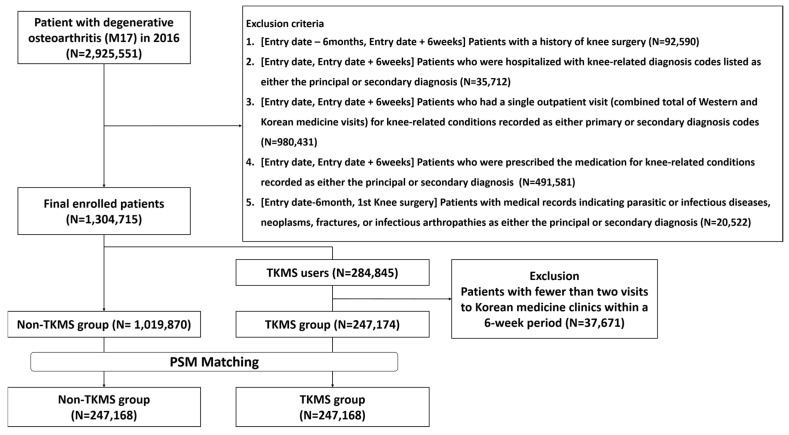
Flowchart of study population selection. According to the 1:1 propensity score matching by age, sex, and Charlson comorbidity index (CCI), 247,168 patients were included in the non-traditional Korean medicine service group and the traditional Korean medicine service group.

In 2016, 2,925,551 patients received medical care for KOA as their primary diagnosis. After applying the exclusion criteria, 1,304,715 remained: 247,174 in the TKMS group and 1,109,870 in the Non-TKMS group. After 1:1 propensity score matching by age, sex, CCI, and the number of outpatient visits over a 6-week period, 494,336 patients (247,168 per group) were included in the final dataset (Figure 2).

After propensity score matching, the SMDs for sex, age, insurance payment type, CCI score, and number of outpatient visits were below 0.1 (Table 1, Table S1), indicating no statistically significant differences between groups.

### 3.2. Comparison of Knee Surgery and Opioid Use by TKMS Use

**Figure 3:**
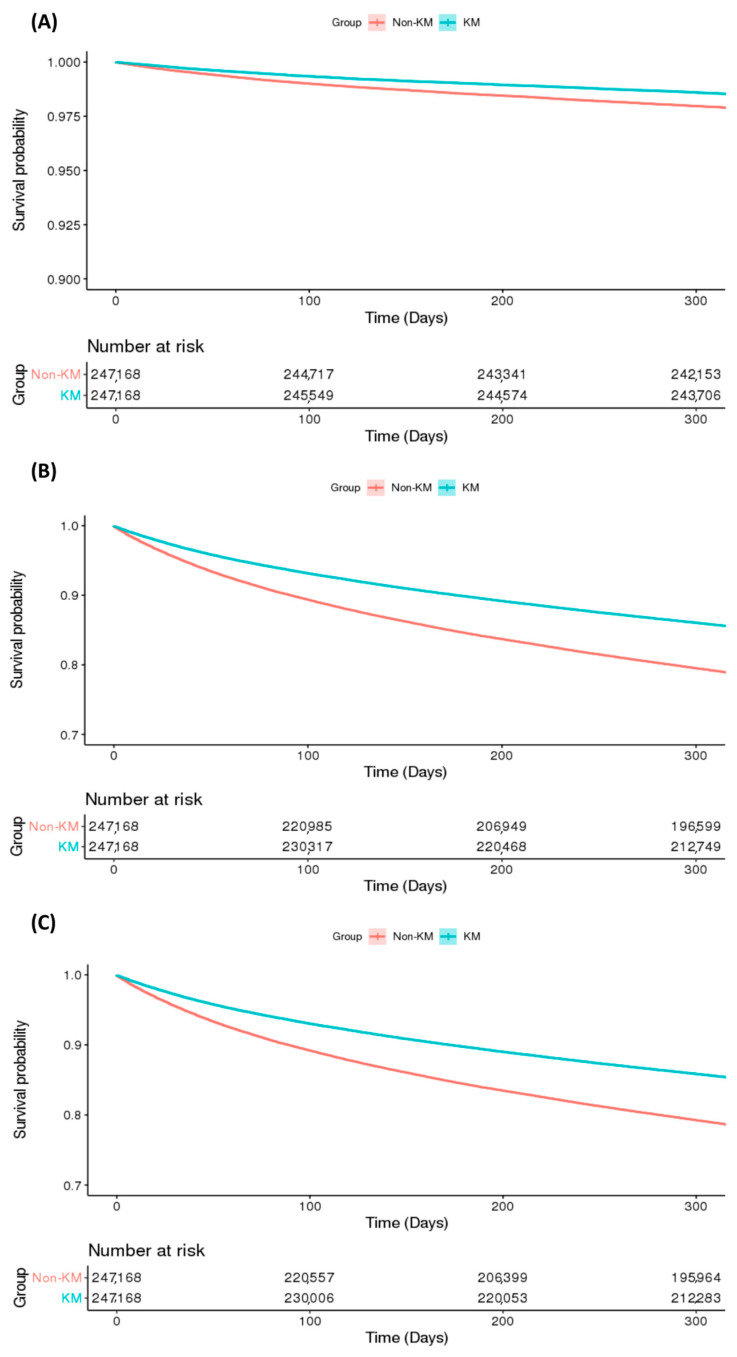
Kaplan–Meier survival estimates for incidence of (**A**) knee surgery, (**B**) opioid use, (**C**) and compound events. KM group denotes the traditional Korean medicine service group.

Kaplan–Meier survival analysis revealed that the TKMS group had a lower cumulative incidence of knee surgery, opioid use, and compound events compared to the Non-TKMS group (log-rank test, *p* < 0.001; Figure 3). Cox proportional hazards modeling confirmed statistically lower HRs in the TKMS group for knee surgery (HR = 0.69; 95% CI: 0.66–0.72), opioid use (HR = 0.65; 95% CI: 0.65–0.66), and compound events (HR = 0.66; 95% CI: 0.65–0.67) compared to the Non-TKMS group (Table 2). The PH assumption was verified using a log–log plot (Figure S1).

### 3.3. Sensitivity Analysis

A sensitivity analysis was conducted by changing the TKMS group’s index date to 4, 8, or 10 weeks after the entry date. In all scenarios, TKMS use lowered the HRs for knee surgery, opioid use, and compound events (Table 3). Survival analyses confirmed significantly lower HRs in the TKMS group compared to the Non-TKMS group for knee surgery, opioid use, and compound events (*p* < 0.001; Tables S3–S5).

## 4. Discussion

This study assessed the impact of TKMS on the incidence of subsequent knee surgery and opioid use in patients with KOA. The TKMS group had a significantly lower risk of knee surgery and opioid use compared to the Non-TKMS group.

This study demonstrated that the utilization of TKMS was associated with a reduced risk of surgery in patients with KOA. Although surgical treatment may be considered when nonsurgical management fails, surgery does not necessarily guarantee improved outcomes. Previous studies have reported variable efficacy between arthroscopic surgery and conservative treatment [26]. Moreover, surgery can increase the risk of falls in patients with KOA [26]. Arthroscopy has been shown to triple the risk of progression to total knee arthroplasty (TKA), and TKA itself is associated with a four-fold increase in complications during the follow-up period [27].

Surgery is also linked to considerable socioeconomic costs. A previous study reported that patients who underwent TKA have comparable medical expenses and income to the control group up to 3 years before surgery but consistently experience higher medical costs and reduced income after surgery. This was attributed to factors like lost earnings from work, requirements for home care, and increased medication use [28]. These reports suggest an increase in direct surgical expenditures and ongoing socioeconomic costs after surgery.

Utilization of TKMS was also associated with a reduced risk of opioid use in patients with KOA. Long-term opioid use has been reported in more than 800,000 patients with KOA in the United States [29]. However, opioids have a higher incidence of side effects than non-opioids, without offering superior efficacy in terms of pain relief or functional improvement [30]. Additionally, in patients with osteoarthritis, opioids can cause gastrointestinal disorders, nausea, vomiting, loss of appetite, dermatologic adverse events, and central nervous system disorders [31]. Considering these safety concerns, a growing emphasis has been placed on effective management of opioid use and exploring alternative treatment options [32]. The current study indicates that TKMS may be an effective alternative treatment strategy for reducing opioid use.

On the other hand, among older adults, who have a high prevalence of KOA, polypharmacy—the use of multiple drugs or those exceeding medical necessity—is common due to multiple underlying conditions [33]. Polypharmacy may lead to issues such as drug–drug interactions, toxicity, falls, injuries, cognitive impairment (such as delirium), and poor adherence to medication regimens [34]. Excessive polypharmacy has been associated with reduced nutritional status, decreased functional ability, and impaired cognitive capacity compared to those not experiencing polypharmacy [35]. Therefore, lowering the risk of opioid use has meaningful implications for addressing polypharmacy-related issues. Moreover, one study reported that acupuncture provided greater pain reduction than NSAIDs in patients with KOA [36], raising the possibility that TKMS use may also be associated with reduced use of non-opioid analgesics such as NSAIDs. Considering that a substantial proportion of patients with chronic pain are exposed to polypharmacy [37], our findings suggest that TKMS use could contribute to mitigating polypharmacy. Further related research is needed.

Acupuncture, a major KM treatment modality, helps manage osteoarthritis pain and prevent functional issues by improving joint blood flow, regulating inflammatory cytokine levels, and activating pain-modulating pathways to reduce the nociceptive response in the central nervous system [38,39]. Recent research also supports the effectiveness of other KM modalities, such as herbal medicine, pharmacopuncture, and Chuna manual therapy, for KOA. Herbal medicines exert chondroprotective effects on the cartilage through their anti-inflammatory and antioxidant actions [40]. Pharmacopuncture combines mechanical stimulation with pharmacological action to relieve pain and restore joint function [41]. Chuna manual therapy can correct misaligned joints and reduce mechanical stress by relieving muscle imbalances [42,43]. The analgesic, anti-inflammatory, and joint function-enhancing effects of these KM modalities provide a plausible explanation for the observed associations between TKMS utilization and the reduced need for surgery and opioid use among patients with KOA.

Randomized controlled trials have reported that acupuncture can alleviate pain and enhance physical function in patients with KOA. An individual patient data meta-analysis examining acupuncture’s efficacy for four chronic pain conditions reported that the effects of acupuncture were sustained over time [44]. Indeed, electroacupuncture and manual acupuncture are reportedly more effective than sham acupuncture in reducing pain and improving function [45]. However, most previous research has focused on short-term and subjective outcomes or measures, with relatively little known about objective and long-term outcomes such as surgery or medication use. Therefore, the value of the present study lies in the use of large-scale NHI claims data to evaluate clinically relevant objective outcomes (effect of TKMS on surgery and opioid use).

This study has several limitations. First, as a retrospective cohort study, the results should be interpreted with caution. In particular, the NHI claims data lack details related to non-reimbursed treatments and specific KM treatment modalities, making it difficult to analyze the efficacy of individual intervention. Treatment frequently used in TKMS such as pharmacopuncture and herbal medicine, are non-reimbursed and thus not captured in claims data. Accordingly, we defined TKMS solely based on visits to KM clinics or hospitals rather than the receipt of specific modalities. Nevertheless, considering that patients in South Korea typically receive integrated TKMS, including acupuncture, pharmacopuncture, and Chuna manual therapy, the study design was deemed suitable for real-world clinical practice.

Second, due to the inherent limitations of claims data, patient-reported outcomes such as pain intensity, functional scores, or quality of life could not be assessed. It is therefore unclear whether the lower incidence of surgery in the TKMS group reflects true symptom improvement or a preference among TKMS users to avoid surgery. Future studies using hospital-based electronic medical records that include non-reimbursed treatment details and patient-reported outcomes are warranted.

Third, we attempted to minimize selection bias between patients who preferred TKMS and those who did not through propensity score matching. However, while this method adjusts for measured confounders, it cannot eliminate the possibility of residual confounding by unmeasured factors [46]. Patient preferences for non-invasive treatments, health literacy, socioeconomic status, and the severity of KOA are potential unmeasured variables that may have influenced the observed association.

Finally, regarding opioid outcomes, our analysis defined opioid use as having received at least one prescription for an opioid medication. Information on treatment duration or cumulative dose was not available. Considering that short-term and long-term opioid use carries very different clinical implications, including the risk of substance abuse [46], a more granular analysis would have provided greater clarity on the role of TKMS in reducing opioid use.

Nevertheless, this is the first study to show that TKMS are associated with lower rates of knee surgery and opioid use among patients with KOA, based on large-scale NHI claims data from HIRA. The main strengths of this study are the use of objective outcomes—surgery and opioid use—evaluated at the national population level, and the consistent hazard ratios across sensitivity analyses with varying treatment windows, which reinforce the robustness of the findings. Further research should include prospective cohort studies and Randomized controlled trials with comprehensive treatment records, patient characteristics, symptom severity, and radiographic evidence.

## 5. Conclusions

The analyses in this study demonstrate that TKMS use in patients with KOA may be associated with reduced subsequent knee surgeries and opioid use. These findings offer insights for clinicians, patients, and policymakers for consideration when evaluating treatment options and developing healthcare policies.

## Figures and Tables

**Table 1:** Baseline characteristics of the propensity score-matched cohorts.

		*n*	Non-TKMS		TKMS		SMD
			*n*	%	*n*	%	
Total N		494,336	247,168	100.0	247,168	100.0	-
Sex	Male	124,083	62,052	25.1	62,031	25.1	0.00
	Female	370,253	185,116	74.9	185,137	74.9	0.00
Age_group	<40	23,623	11,806	4.8	11,817	4.8	0.00
	40–49	39,746	19,889	8.1	19,857	8.0	0.00
	50–59	99,842	49,921	20.2	49,921	20.2	0.00
	60–69	144,042	72,021	29.1	72,021	29.1	0.00
	70–79	141,196	70,547	28.5	70,649	28.6	0.00
	≥80	45,887	22,984	9.3	22,903	9.3	0.00
Payer type	NHI	494,336	247,168	100.0	247,168	100.0	-
CCI_group	0	321,832	160,886	65.1	160,946	65.1	0.00
	1	109,688	54,879	22.2	54,809	22.2	0.00
	≥ 2	62,816	31,403	12.7	31,413	12.7	0.00
Total outpatient N	2–5	293,374	146,687	59.4	146,687	59.4	0.00
	6–11	142,445	71,247	28.8	71,198	28.8	0.00
	≥12	58,517	29,234	11.8	29,283	11.9	0.00
F/u surgery survival event	No	485,359	241,862	97.9	243,497	98.5	−0.05
	Yes	8977	5306	2.2	3671	1.5	0.05
F/u opioid use survival event	No	405,320	194,298	78.6	211,022	85.4	−0.18
	Yes	89,016	52,870	21.4	36,146	14.6	0.18
F/u surgery or opioid use survival event	No	404,195	193,657	78.4	210,538	85.2	−0.18
	Yes	90,141	53,511	21.7	36,630	14.8	0.18

TKMS, traditional Korean medicine service; CCI, Charlson comorbidity index; F/U, follow-up; SMD, standardized mean difference; NHI, National Health Insurance.

**Table 2:** Hazard ratios of knee surgery, opioid use, and compound events by traditional Korean medicine service use.

Variable	Knee Surgery		Opioid Use		Knee Surgery or Opioid Use	
	HR (95% CI)	*p*-Value	HR (95% CI)	*p*-Value	HR (95% CI)	*p*-Value *
TKMS use (ref. Non-TKMS)						
KM	0.69 (0.66–0.72)	<0.0001	0.66 (0.65–0.66)	<0.0001	0.66 (0.65–0.67)	<0.0001
**Multivariate model**						
TKMS use (ref. Non-TKMS)						
KM	0.69 (0.66–0.72)	<0.0001	0.65 (0.64–0.66)	<0.0001	0.65 (0.64–0.66)	<0.0001
Sex (ref. Male)						
Female	1.25 (1.18–1.31)	<0.0001	1.23 (1.21–1.25)	<0.0001	1.22 (1.20–1.24)	<0.0001
Age (ref. <40)						
40–49	1.11 (0.96–1.28)	0.1598	1.61 (1.51–1.71)	<0.0001	1.59 (1.49–1.69)	<0.0001
50–59	1.33 (1.17–1.50)	<0.0001	2.51 (2.37–2.66)	<0.0001	2.44 (2.31–2.58)	<0.0001
60–69	1.48 (1.30–1.67)	<0.0001	3.29 (3.11–3.48)	<0.0001	3.15 (2.98–3.33)	<0.0001
70–79	1.66 (1.47–1.88)	<0.0001	4.16 (3.92–4.40)	<0.0001	3.95 (3.74–4.17)	<0.0001
≥80	0.79 (0.67–0.91)	0.0013	4.20 (3.96–4.45)	<0.0001	3.97 (3.75–4.20)	<0.0001
CCI (ref. 0)						
1	0.91 (0.86–0.95)	0.0002	1.10 (1.08–1.12)	<0.0001	1.10 (1.08–1.11)	<0.0001
2	0.58 (0.54–0.62)	<0.0001	1.07 (1.05–1.09)	<0.0001	1.06 (1.04–1.08)	<0.0001
Total outpatient N (ref. 2–5)						
6–11	1.56 (1.49–1.63)	<0.0001	1.31 (1.29–1.33)	<0.0001	1.32 (1.30–1.34)	<0.0001
≥12	1.62 (1.52–1.72)	<0.0001	1.34 (1.32–1.37)	<0.0001	1.35 (1.32–1.38)	<0.0001

* *p*-value from Cox regression analysis adjusted for age, sex, and Charlson comorbidity index. TKMS, traditional Korean medicine service; N, number; CI, confidence interval; HR, hazard ratio; CCI, Charlson comorbidity index.

**Table 3:** Sensitivity analyses according to the length of the treatment assessment window.

	TKMS Use		Non-TKMS			
	Matched Number	Event	Matched Number	Event	HR (95%CI)	*p*-Value *
	*n*	*n* (%)	*n*	*n* (%)		
Knee surgery						
4 weeks	235,249	3765	235,249	5493	0.68 (0.66–0.71)	<0.0001
8 weeks	255,971	3601	255,971	5063	0.71 (0.68–0.74)	<0.0001
10 weeks	262,747	3438	262,747	4833	0.71 (0.68–0.74)	< 0.0001
Opioid use						
4 weeks	235,249	36,471	235,249	53,185	0.65 (0.64–0.66)	<0.0001
8 weeks	255,971	35,429	255,971	52,005	0.65 (0.64–0.66)	<0.0001
10 weeks	262,747	34,348	262,747	50,332	0.65 (0.64–0.66)	<0.0001
Surgery or opioid use						
4 weeks	235,249	36,975	235,249	53,876	0.65 (0.64–0.66)	<0.0001
8 weeks	255,971	35,890	255,971	52,616	0.65 (0.64–0.66)	<0.0001
10 weeks	262,747	34,799	262,747	50,900	0.65 (0.65–0.66)	<0.0001

* *p*-value from Cox regression analysis adjusted for age, sex, and Charlson comorbidity index; HR, hazard ratio; TKMS, traditional Korean medicine service.

## Data Availability

These data are not available publicly. Data used in this study were obtained from the Health Insurance Review and Assessment Service of Korea. To protect patient privacy, access to data was limited to certified researchers within South Korea, analysis was permitted only within the HIRA system, and data export was strictly prohibited.

## Supplementary Materials

The following supporting information can be downloaded at https://www.mdpi.com/article/10.3390/jcm14207152/s1, Figure S1: Log-log plot for 6-week analysis: (A) for knee surgery, (B) for opioids use, (C) for knee surgery or opioids use.; Table S1: Procedure codes for knee surgery included in the analysis; Table S2: Baseline characteristics (before matched); Table S3: The incidence of events during the 4-week treatment period; Table S4: Incidence of events during the 8-week treatment period; Table S5: Incidence of events during the 10-week treatment period.

## Abbreviations

The following abbreviations are used in this manuscript:

CCI	Charlson comorbidity index
CI	Confidence interval
HIRA	Health Insurance Review and Assessment Service
HR	Hazard ratio
KM	Korean medicine
KOA	Knee osteoarthritis
PH	Proportional hazard
PSM	Propensity score matching
SMD	Standardized mean difference
TKA	Total knee arthroplasty
