# Supplementary material for: Traditional Korean Medicine Services and Its Association with Knee Surgery and Opioid Use in Patients with Knee Osteoarthritis: A Nationwide Retrospective Study in Korea
Source: J Clin Med. 2025 Oct 10;14(20):7152. doi: 10.3390/jcm14207152 (PMC12565209; doi:10.3390/jcm14207152)
Supplement: Supplementary file 1 [file jcm-14-07152-s001.zip › jcm-3857016-supplementary.pdf]

Table S1. Procedure codes for knee surgery included in the analysis.

No	Procedure Code	Procedure Name
1	N2072	Replacement Total Arthroplasty
2	N2077	
3	N2712	Replacement Hemiarthroplasty
4	N2717	
5	N3712	Revision of Replacement Arthroplasty
6	N3717	
7	N3722	
8	N3727	
9	N4712	
10	N4717	
11	N4722	
12	N4727	
13	N0702	Excision of Joint
14	N0707	
15	N0722	Resection Arthroplasty
16	N0732	Arthrodesis
17	N0821	Meniscectomy
18	N0826	
19	N0822	
20	N0827	
21	N0823	Repair of Meniscus
22	N0828	
23	N0824	
24	N0829	
25	N0825	Meniscus Allograft Transplantation
26	N0820	
27	N0691	
28	N0692	
29	N0694	
30	N0693	
31	N0695	
32	N0880	Reconstruction and Repair of Cruciate Ligament
33	N0881	
34	N0890	

Table S2. Baseline characteristics (before matched).

		Total N	Non-KM		KM		SMD
			N	%	N	%	
Total N		1,267,044	1,019,870	100.0	247,174	100.0	-
Sex	Male	348,074	286,043	28.1	62,031	25.1	0.07
	Female	918,970	733,827	72.0	185,143	74.9	-0.07
Age_group	< 40	32,962	21,139	2.1	11,823	4.8	-0.15
	40-49	80,727	60,870	6.0	19,857	8.0	-0.08
	50-59	294,931	245,010	24.0	49,921	20.2	0.09
	60-69	415,323	343,302	33.7	72,021	29.1	0.10
	70-79	333,392	262,743	25.8	70,649	28.6	-0.06
	≥ 80	109,709	86,806	8.5	22,903	9.3	-0.03
Payer type	NHI	1,267,044	1,019,870	100.0	247,174	100.0	-
CCI_group	0	807,759	646,808	63.4	160,951	65.1	-0.04
	1	290,651	235,841	23.1	54,810	22.2	0.02
	≥ 2	168,634	137,221	13.5	31,413	12.7	0.02
Total outpatient N	2-5	1,002,503	855,816	83.9	146,687	59.4	0.57
	6-11	200,166	128,968	12.7	71,198	28.8	-0.41
	≥ 12	64,375	35,086	3.4	29,289	11.9	-0.32
F/u surgery survival event	No	1,242,879	999,376	98.0	243,503	98.5	-0.04
	Yes	24,165	20,494	2.0	3,671	1.5	0.04
F/u opioid use survival event	No	405,320	194,298	78.6	211,022	85.4	-0.18
	Yes	89,016	52,870	21.4	36,146	14.6	0.18
	No	404,195	193,657	78.4	210,538	85.2	-0.18
	Yes	90,141	53,511	21.7	36,630	14.8	0.18

KM, Korean medicine; Non-KM, Non-Korean medicine; N, number; CCI, Charlson comorbidity index; F/U, follow-up; SMD, standardized mean difference; NHI, National Health Insurance.

Table S3. The incidence of events during the 4-week treatment period.

Event	Group	Total	Event	Censored	*p*-Value for Log-Rank Test
Knee surgery	KM	235,249	3,765	231,484	<.0001
	Non-KM	235,249	5,493	229,756	
	Total	470,498	9,258	461,240	
Opioids use	KM	235,249	36,471	198,778	<.0001
	Non-KM	235,249	53,185	182,064	
	Total	470,498	89,656	380,842	
Knee surgery or opioids use	KM	235,249	36,975	198,274	<.0001
	Non-KM	235,249	53,876	181,373	
	Total	470,498	90,851	379,647	

KM, Korean medicine; Non-KM, Non-Korean medicine.

Table S4. Incidence of events during the 8-week treatment period.

Event	Group	Total	Event	Censored	*p*-Value for Log-Rank Test
Knee surgery	KM	255,971	3,601	252,370	<.0001
	Non-KM	255,971	5,063	250,908	
	Total	511,942	8,664	503,278	
Opioids use	KM	255,971	35,429	220,542	<.0001
	Non-KM	255,971	52,005	203,966	
	Total	511,942	87,434	424,508	
Knee surgery or opioids use	KM	255,971	35,890	220,081	<.0001
	Non-KM	255,971	52,616	203,355	
	Total	511,942	88,506	423,436	

KM, Korean medicine; Non-KM, Non-Korean medicine.

Table S5. Incidence of events during the 10-week treatment period.

Event	Group	Total	Event	Censored	*p*-value for Log-Rank Test
Knee surgery	KM	262,747	3,438	259,309	<.0001
	Non-KM	262,747	4,833	257,914	
	Total	525,494	8,271	517,223	
Opioids use	KM	262,747	34,348	228,399	<.0001
	Non-KM	262,747	50,332	212,415	
	Total	525,494	84,680	440,814	
Knee surgery or opioids use	KM	262,747	34,799	227,948	<.0001
	Non-KM	262,747	50,900	211,847	
	Total	525,494	85,699	439,795	

KM, Korean medicine; Non-KM, Non-Korean medicine.

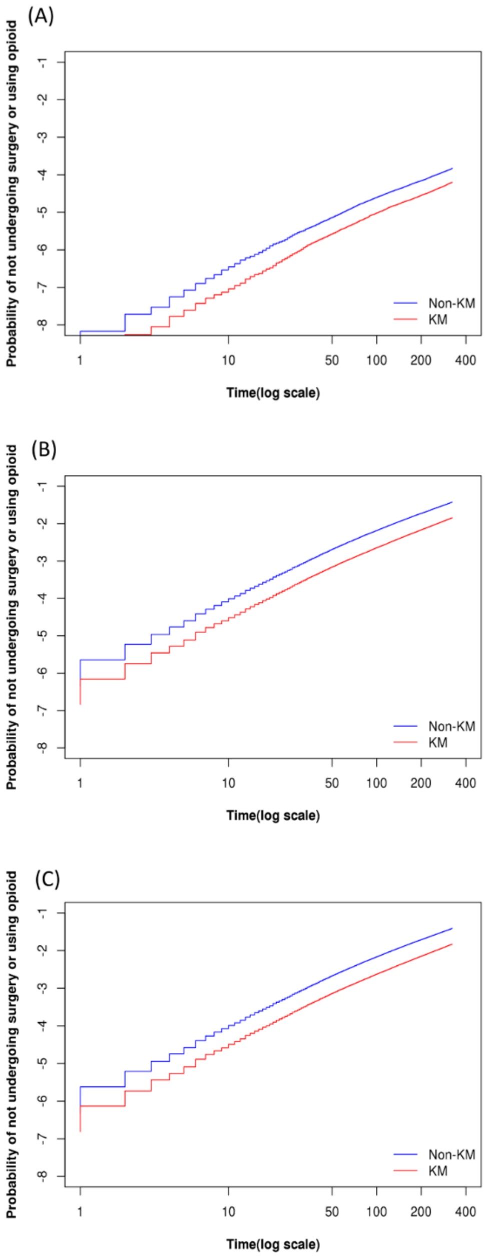

Figure S1. Log-log plot for 6-week analysis: (A) for knee surgery, (B) for opioids use, (C) for knee surgery or opioids use.
